# Supplementary figures and images for: Progranulin deficiency leads to reduced glucocerebrosidase activity
Source: PLoS One. 2019 Jul 10;14(7):e0212382. doi: 10.1371/journal.pone.0212382 (PMC6619604; doi:10.1371/journal.pone.0212382)

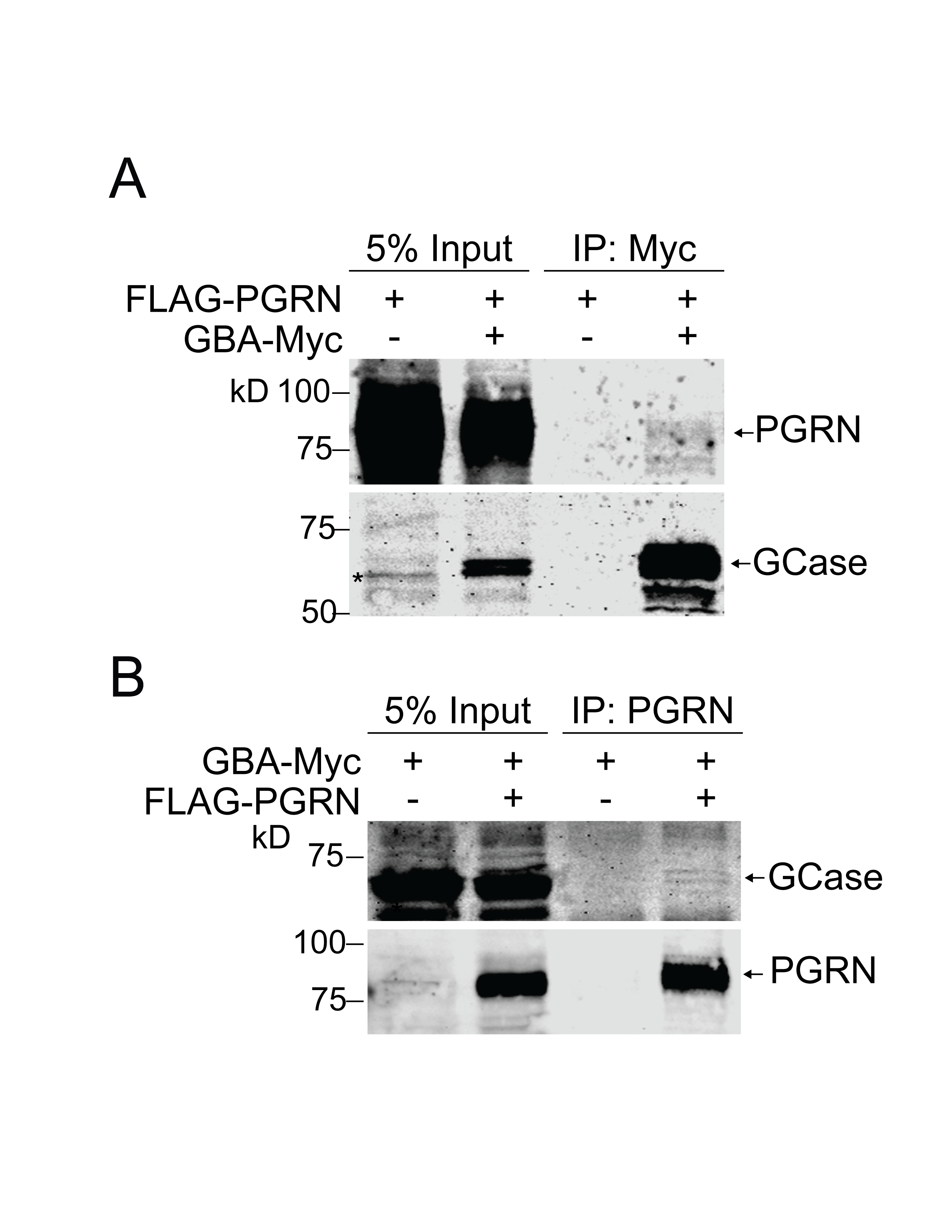

Supplement: S1 Fig — HEK293T cells were transfected with mGBA-FLAG-myc and FLAG-mPGRN constructs as indicated and anti-myc (A) or anti-PGRN (B) immunoprecipitation experiments were carried out. The presence of PGRN and GCase in the immunoprecipitates were detected using Western blot analysis. (TIF) [file pone.0212382.s001.tif]

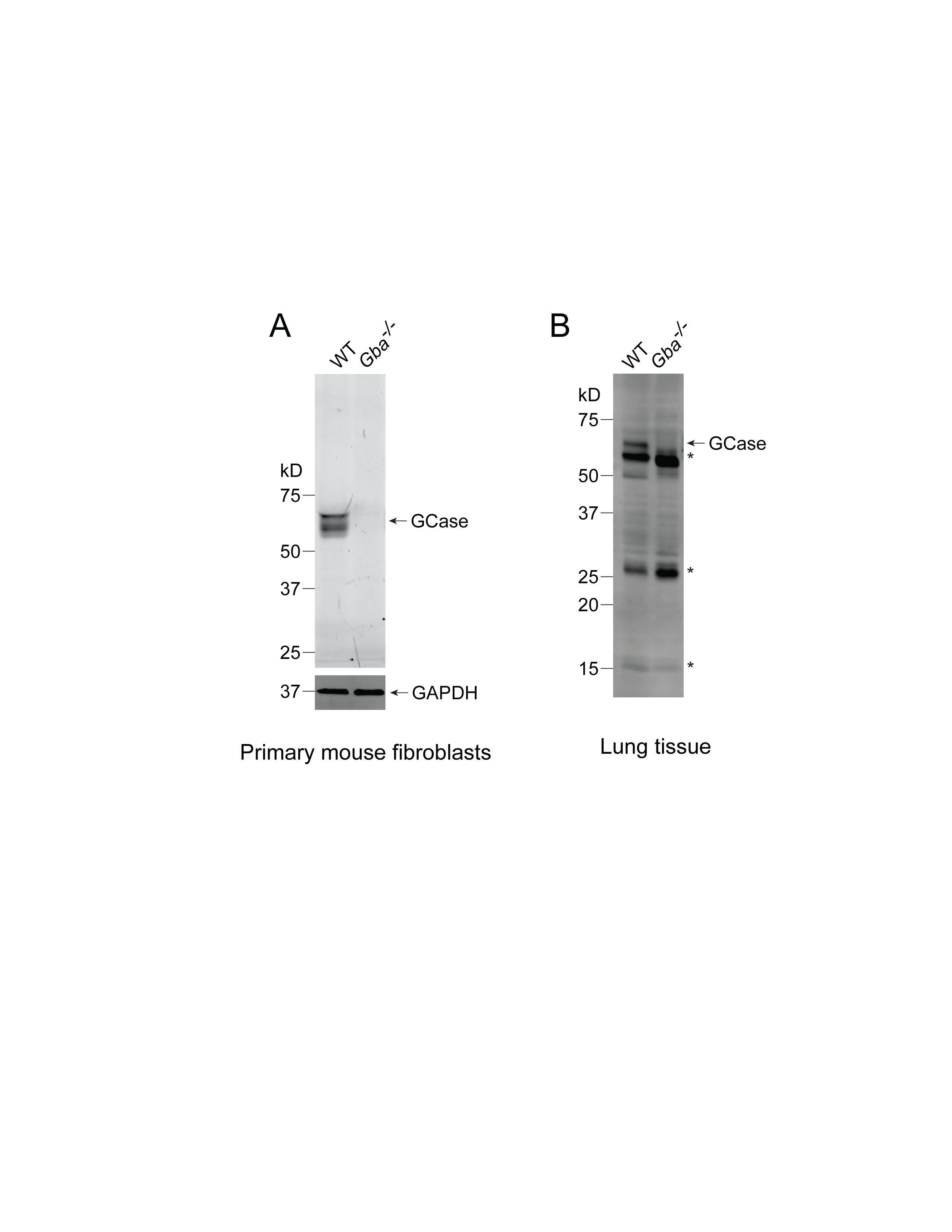

Supplement: S2 Fig — A) Primary fibroblasts from WT and Gba-/- mice. B) Lung tissue lysates from WT and Gba-/- mice. *indicates non-specific bands. (TIF) [file pone.0212382.s002.tif]

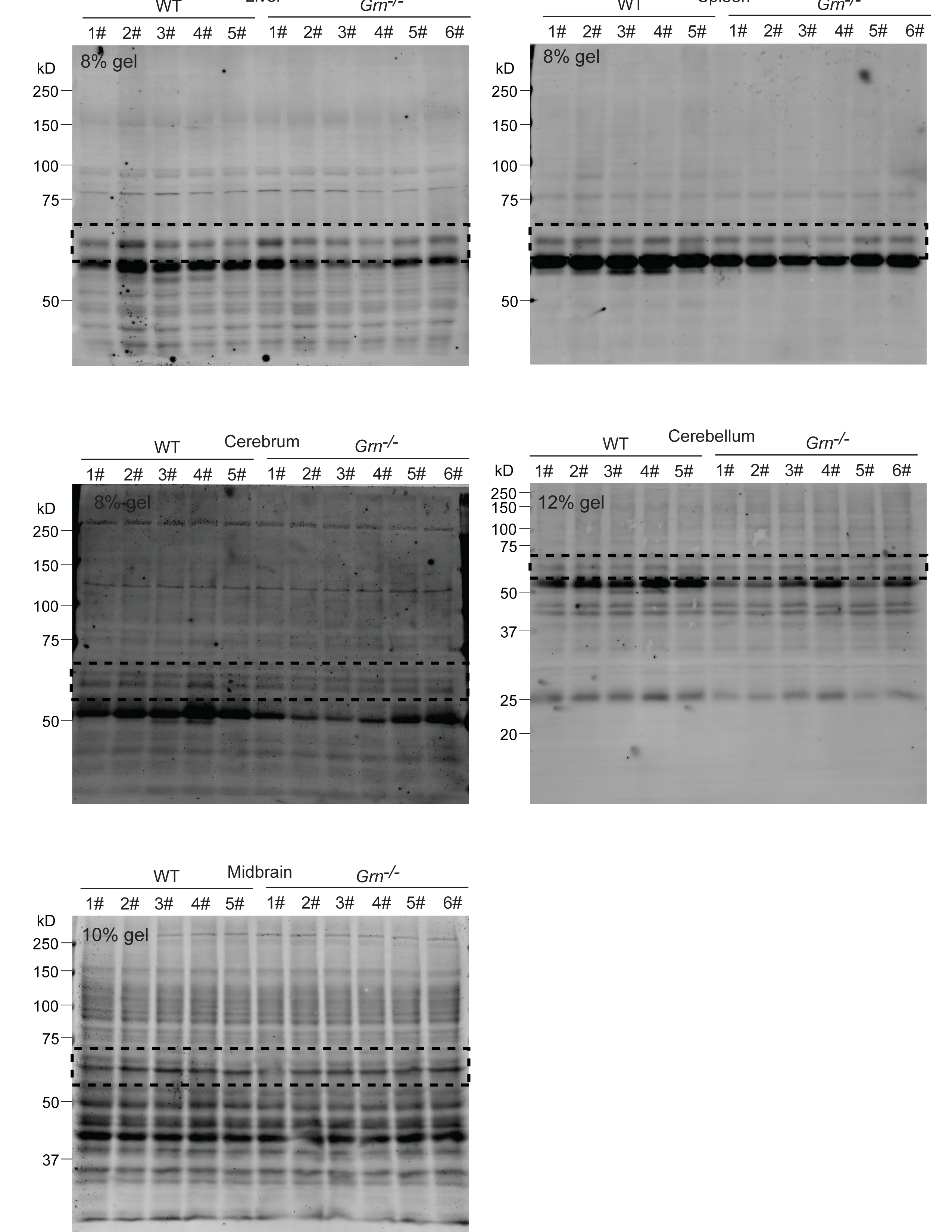

Supplement: S3 Fig — Dashed line indicated where the GCase bands are. (TIF) [file pone.0212382.s003.tif]

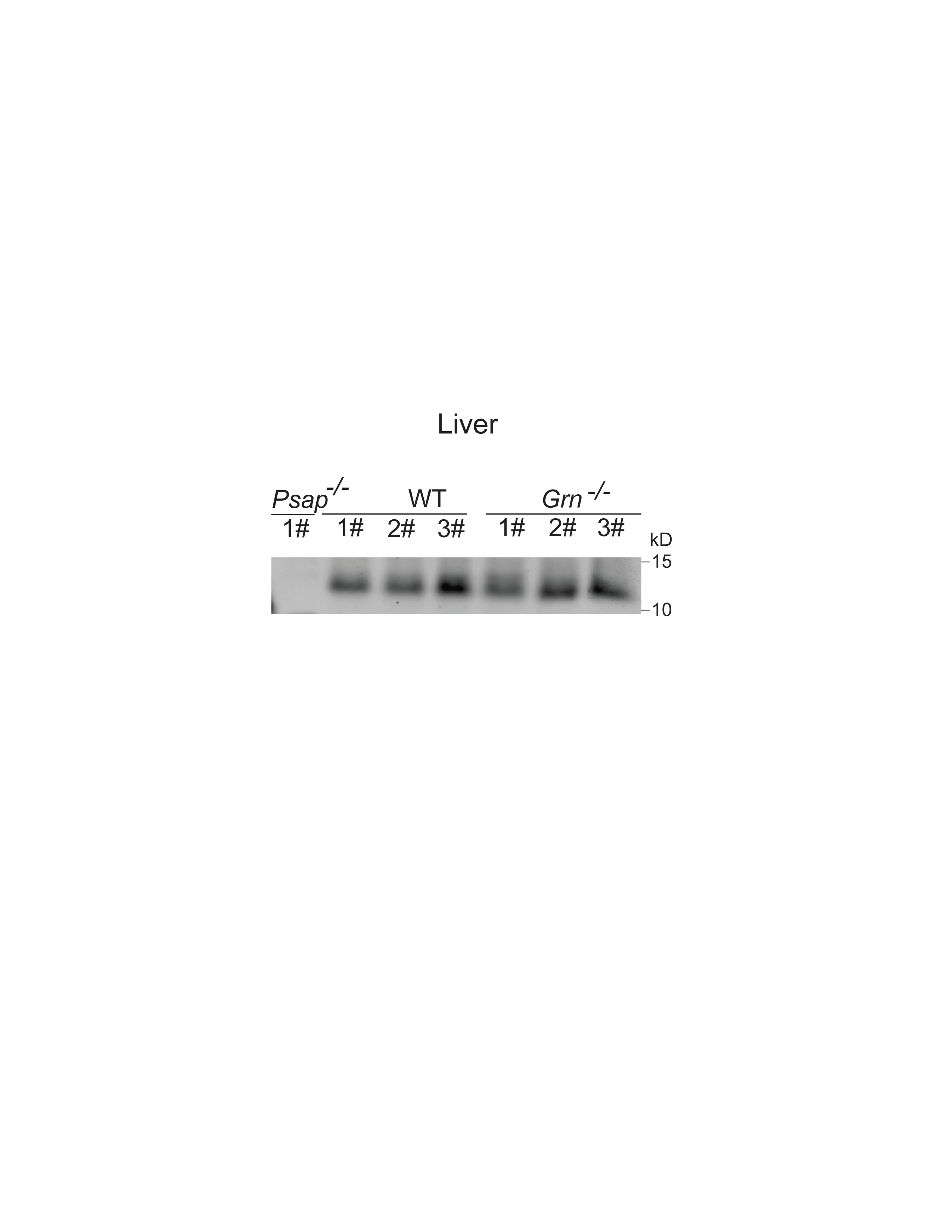

Supplement: S4 Fig — WT and PGRN-deficient liver lysates were immunoprecipitated using anti-saposin C antibodies and the IP products were analyzed by Western blot using polyclonal anti-PSAP antibodies. (TIF) [file pone.0212382.s004.tif]

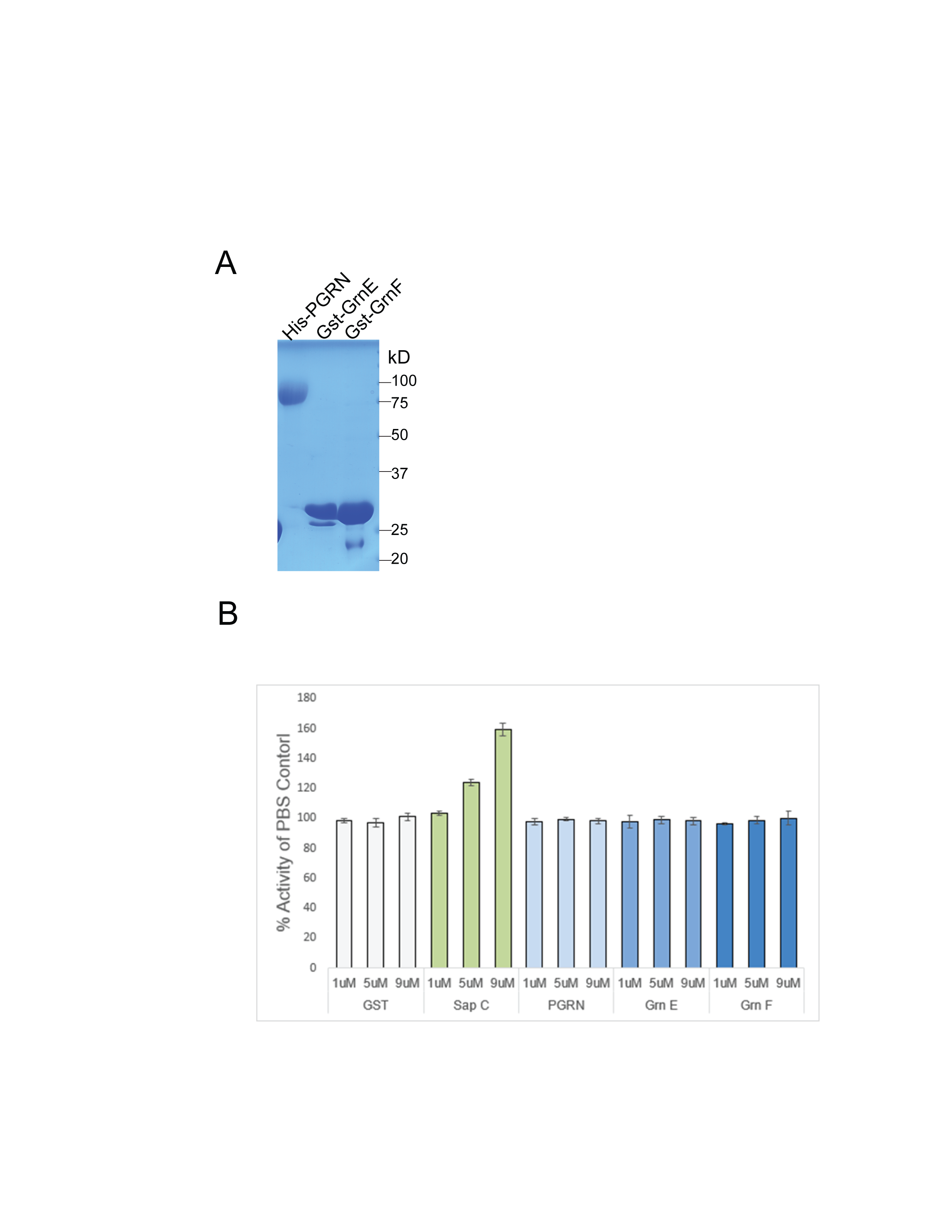

Supplement: S5 Fig — (A) Commassie staining of recombinant PGRN, Grn E and Grn F proteins used in the activity assay. (B) Activity of recombinant GCase (Cerezyme) was measured with the addition of recombinant PGRN, Grn E, Grn F, or recombinant saposin C as a positive control. (TIF) [file pone.0212382.s005.tif]

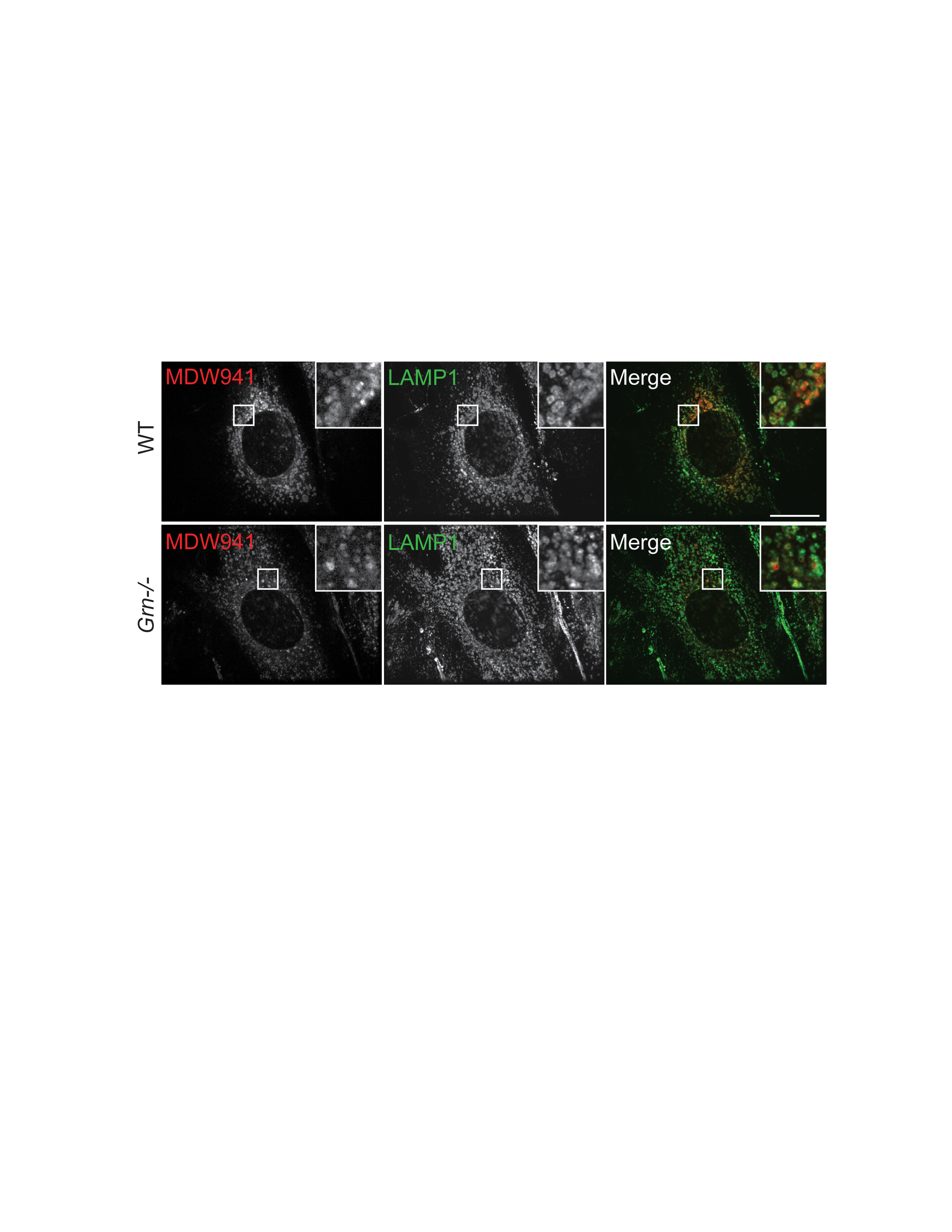

Supplement: S6 Fig — WT and Grn-/- fibroblasts were labeled for 2 hours with MDW941 before fixation and immunostaining. Scale bar = 20 μm. (TIF) [file pone.0212382.s006.tif]
